# Potential of RAS Inhibition to Improve Metabolic Bone Disorders

**DOI:** 10.1155/2013/932691

**Published:** 2013-07-22

**Authors:** Yoseph Gebru, Teng-Yue Diao, Hai Pan, Emmanuel Mukwaya, Yan Zhang

**Affiliations:** ^1^Center for Systems Biomedical Sciences, University of Shanghai for Science and Technology, Shanghai 200093, China; ^2^School of Medical Instrument and Food Engineering, University of Shanghai for Science and Technology, Shanghai 200093, China; ^3^Department of Applied Biology and Chemical Technology, The Hong Kong Polytechnic University, Hung Hom, Kowloon, Hong Kong

## Abstract

Metabolic bone disorder is usually caused by abnormalities of minerals and hormones metabolism. Recently, it has been proved by several studies that the renin-angiotensin system (RAS) in local bone tissue is directly involved in bone metabolism. Activation of skeletal RAS plays an important role in bone metabolic disorders. Based on *in vitro*, *in vivo*, and clinical studies, this review explains the roles of RAS in bone metabolism and also covers the potential approaches and beneficial effects of RAS inhibition on bone health. Differential strategies for inhibiting RAS can be employed to maintain bone health, which are attributed primarily to the reduced level of angiotensin II (AngII) and suppressed stimulation of the AngII signaling pathway. The use of renin inhibitors, angiotensin-converting enzyme inhibitors, and AngII receptor blockers either individually or in combination with each other could have promising results in fighting bone metabolic disorders associated with other cardiovascular diseases as well as independent bone injuries.

## 1. Introduction

Metabolic bone disorder is a common pathological mechanism with genetic components characterized by reduced bone mass and increased risk of fractures [1]. It is usually caused mainly by abnormalities of minerals such as calcium, phosphorus, and hormones like vitamin D and parathyroid hormone (PTH). Moreover, there are several physiological mechanisms that are able to cause bone disorders through different molecular pathways including the renin angiotensin system (RAS) which will be explained in this paper. It is a well-established finding that the integrity and resistance of bone depend upon the balance between bone formation by osteoblasts and bone resorption by osteoclasts. Therefore, any biological mechanism that increases osteoclast number and their ability to induce bone absorption or decreases osteoblast number and their ability to form bone may trigger bone disorders. The RAS has been reported to be among these mechanisms that play an important role  in the pathogenesis and progression of metabolic bone disease through different ways [2].

The first step of the RAS, namely, the conversion of angiotensinogen to angiotensin I (AngI) is activated by renin, a highly selective protease secreted from the juxtaglomerular cells in the kidneys. AngI is then converted to angiotensin II (AngII) by the action of angiotensin-converting enzyme (ACE). The relation between the RAS and bone metabolism is mainly based on the regulation of AngII on bone metabolism. Previous studies have reported that AngII significantly increased TRAP-positive multinuclear osteoclasts with the upregulation of RANKL expression through extracellular kinase of osteoblast [3]. These effects were abolished with cotreatment by ACE inhibitors or angiotensin type 1 receptor blockers (ARBs).**  **These findings are very helpful in targeting RAS as another strategy to treat metabolic bone disorders like osteoporosis.

Several studies have been performed to evaluate the beneficial effects of some RAS-targeting drugs on the quality of bone mainly RAS-inhibiting drugs. Drugs that inhibit the RAS, namely, angiotensin-converting enzyme inhibitors (ACE-I) and angiotensin receptor blockers (ARBs) are gaining increasing attention to evaluate their potential to increase bone quality and decrease fractures mainly in bone disorders associated with diabetes. Cross-sectional studies on elderly Chinese populations as well as hypertensive menopausal women and a prospective cohort study on older American men showed an association of ACEIs use with higher bone mineral density [4]. Additionally, direct renin inhibitors can also have more potential for inhibiting the pathway with higher extent. Recent epidemiological studies have reported the benefit of these drugs on increasing bone mass and decreasing the risk for bone fractures [3]. This paper will review the possible mechanisms by which RAS is involved in bone metabolic disorder and the *in vitro* and *in vivo *studies supporting the idea that RAS inhibition can improve bone quality and reduce bone fractures. 

## 2. The Relation between the RAS Cascade and Bone Metabolism

Because the vascular system plays an important role in bone remodeling, the effect of the RAS on bone metabolism was considered to be related only to the regulation of blood flow. However, recent studies are providing some evidences for a direct relation of the renin-angiotensin system with bone metabolism. Most of these studies indicate that activation of the RAS causes abnormal bone metabolism mainly through an elevation of osteoclastic bone resorption, by using a transgenic mouse model overproducing human renin and angiotensinogen or an infusion of AngII in ovariectomized rats [5]. AngII, the dominant effector peptide of the RAS, regulates several cellular mechanisms in a wide variety of tissues in pathobiological states including bone tissue in association with AngII type 1 receptor (AT1) [6]. In a study that examined the effect of AngII on the differentiation of rat calvarial osteoblastic cells, it showed that AngII inhibited mRNA expression of osteocalcin (a protein specifically expressed during maturation of osteoblastic cells) and decreased the activity of alkaline phosphatase (a marker of osteoblastic differentiation) via its receptor AT1 [7].

### 2.1. Circulatory System RAS

The differentiation of osteoblast and osteoclasts is primarily controlled by two key mediators, core-binding factor subunit alpha-1 (Cbfa1) and receptor activator of nuclear factor kappa-B ligand (RANKL), which is regulated by the second messenger, cyclic adenosine monophosphate (cAMP) [8]. cAMP is a key intracellular signaling molecule in controlling bone homeostasis, and its levels in plasma and urine were elevated both in osteoporotic and hypertensive patients who apparently have activated RAS [6]. This indicates that the circulating AngII stimulates an increase of intracellular cAMP and then activates downstream signaling pathways which in turn alter Cbfa1 expression [9]. It is thus plausible that AngII alters the expression of Cbfa1 and subsequently reduces osteoblast number to cause impaired bone formation by activating the cAMP signaling pathway. Opposite to the role of AngII on Cbfa1, the expression of RANKL in osteoblasts, a marker for osteoclastic activation, is significantly increased by AngII [10]. 

In another study which used a chimeric RAS model of transgenic THM (Tsukuba hypertensive mouse) expressing the human renin and human angiotensinogen genes, a recent study showed that the activation of RAS induces high turnover osteoporosis. Here, AngII acted on osteoblasts and not directly on osteoclast precursor cells and increased osteoclastogenesis-supporting cytokines, RANKL, and vascular endothelial growth factor, thereby stimulating the formation of osteoclasts [11]. It has also been suggested that AngII acts on bone cells by binding to AT1 receptors on osteoblasts thereby promoting the release of mediators. In addition, circulating AngII may also influence calcium metabolism by decreasing ionized calcium and increasing parathyroid hormone levels [12]. This is supported by the evidence that AngII shows the ability to inhibit the expression of osteocalcin and decrease the activity of alkaline phosphatase, both of which are essential for bone matrix synthesis and maturation and regulated by Cbfa1 [13].

### 2.2. Local Tissue RAS

It has been recognized that local tissue RAS plays an important role in bone metabolism independent from the systemic involvement of RAS supported by several evidences. Local tissue-specific RAS has been identified to regulate regeneration, cell growth, apoptosis, inflammation, and angiogenesis. It is clear that the RAS is regarded as a local tissue system within bone tissues and bone marrow as the components of the RAS are expressed in osteoblasts and osteoclasts [14]. The association of renin, angiotensin-converting enzyme (ACE) and AngII and its AT1 and AT2 receptors with both normal and disturbed haematopoiesis can be an evidence for the existence of a local bone marrow RAS [15]. The expression of the mRNAs of these major RAS components of human bone marrow samples has also been quantified by reverse transcription-polymerase chain reaction (RT-PCR) to confirm the presence of the local bone marrow RAS [6]. The expression of RAS components by rat unfractionated bone marrow cells (BMCs), haematopoietic-lineage BMC, and cultured marrow stromal cells was also investigated to determine which specific cell types may contribute to a local bone marrow RAS [16].

Considering that RAS can be activated in local bone marrow tissue, these findings lead to a hypothesis and further study about the role of local RAS on bone metabolism. Our group has demonstrated the mRNA expression of RAS components in mice tibia for the first time, and this local bone RAS is involved in age-related osteoporosis and renal osteodystrophy induced by acute kidney disease [17, 18]. Recently, the involvement of local RAS in the pathology of bone disease was proved by treating mice with losartan, where losartan improved hypertension but exacerbated osteopenic phenotype [11]. Therefore, it can be concluded that AngII accompanied by its receptors act on bone cells via a tissue RAS that regulates osteoclast differentiation and affect bone metabolism [12].

## 3. RAS Inhibition to Ameliorate Bone Metabolic Disorders

As activation of the RAS stimulates the expression of osteoclastogenic cytokines in osteoblasts, thereby leading to a high turnover bone disease, it is reasonable to hypothesize that blockade of the RAS may reduce these symptoms. It should also be noted that inhibition of the RAS improves bone quality independently of its effect on blood pressure. This was proved by the cross-talk between AT1 and AT2 receptors of AngII which will be discussed below. The RAS cascade can be interrupted at different stages of the pathway and can be used as a strategy to improve bone quality and prevent further fractures [19].

The core purpose of RAS inhibition is to decrease the production of AngII thereby reducing the adverse effects of its actions on bone deterioration. Three critical steps in the RAS pathway can be targeted for administering specific inhibitor drugs. The first and rate-limiting step of RAS, namely, the conversion of angiotensinogen to AngI is activated by renin, and renin inhibitors can have a great potential in reducing actions of AngII on bone. Aliskiren is a drug of the second generation of renin inhibitors, and it binds to the S3^ bp^ binding site of renin essential for its activity thereby inhibiting its activity [20].

The other target step for RAS inhibitors on the pathway is the conversion of AngI to AngII by ACE, in which ACE-inhibitor drugs are employed. The third target for specific blockage is inhibiting the pathway of AngII signaling by antagonizing AT1 which is the main cell surface receptor for AngII as well as blockage of AT2. Blockage of AT1 can be achieved by administering Angiotensin II receptor blockers (ARBs). Moreover, it should be noted that RAS inhibition and blockage can be achieved through applying specific drugs that act on particular steps of the cascade or using these methods in combination. Renin inhibitors, ACE inhibitors, and ARBs are the most important treatment methods being applied currently for *in vivo* and *in vitro,* as well as clinical research and treatments. 

### 3.1. *In Vitro* Studies

Previous *in vitro* studies have suggested that RAS may be involved in the regulation of bone cells, even though it was not known whether molecules involved in RAS are present in bone *in vivo*. AngII stimulates DNA and collagen synthesis and decreases alkaline phosphatase activity on bone cell populations derived from the periosteum of fetal rat calvariae and on human adult bone cells obtained by collagenase digestion from trabecular bone [21]. It is known that the receptors of AngII are expressed in culture osteoclasts and osteoblasts, and AngII is postulated to be able to act upon the cells involved in bone metabolism [22]. 

In another previous study, AngII stimulated bone resorption in cocultures of osteoclasts with osteoblastic cells, and other bone cells and this action was inhibited by using ACE inhibitors [7]. But it showed no effect either on osteoclast formation or on bone resorption by isolated osteoclasts on this study. In bone marrow-derived mononuclear cells, AngII significantly increased tartrate-resistant-acid-phosphatase- (TRAP-) positive multinuclear osteoclasts [10]. In addition, AngII significantly induced the expression of receptor activator of NF-*κ*B ligand (RANKL) in osteoblasts, leading to the activation of osteoclasts, whereas these effects were completely blocked by an AngII type 1 receptor blocker (olmesartan) and mitogen-activated protein kinase kinase inhibitors. Therefore, it can be suggested that AngII acts on osteoclasts through the signals and cellular communication between osteoblasts and osteoclasts.

### 3.2. *In Vivo* Studies

Recent studies have also demonstrated the expression of RAS components in osteoblasts and osteoclasts *in vivo* [16]. To find out whether *in vivo* bone cells are the targets of AngII, recent studies have examined the expression of AngII receptor proteins, ACE, and renin in the bone of adult mice. It has been shown that ACE, AT1, and AT2 are expressed in osteoclasts and osteoblasts, and renin is expressed neither by osteoclasts nor by osteoblasts but by cells within the bone microenvironment [22]. 

Studies have shown that excessive activation of RAS causes osteoporosis, mainly through an elevation of osteoclastic bone resorption, by using a transgenic mouse model overproducing human renin and angiotensinogen or an infusion of AngII in ovariectomized rats. Several recent *in vivo* studies have reported that inhibition of the RAS at different stages have a crucial beneficial effect in combating the adverse effects of bone mineral disorders. Ovariectomy (OVX) rat models have shown a significant increase in osteoclast activation as assessed by the tartrate-resistant acid phosphatase (TRAP) activity in the tibia and a significant decrease in bone density evaluated by dual-energy X-ray absorptiometry. This OVX-induced decrease in bone density and increase in TRAP activity were attenuated by the treatment with an ACE inhibitor, Imidapril [10].

In a recent study, mice lacking the gene encoding the major AngII receptor isoform, AngII type 1A receptor (AT1a), were studied using micro CT scanning, histomorphometric, and biochemical techniques. Both male and female AT1a knockout mice exhibited an increased trabecular bone volume, trabecular bone number, and connectivity at tibial metaphysis. Quantitative RT-PCR using RNA isolated from the tibia and femur revealed that the RANKL/osteoprotegerin (OPG) ratio was increased [11]. Another study which investigated the effects of AT2 receptor blocker on bone mass revealed that AT2 receptor as well as renin and ACE were expressed in bone and that AT2 receptor blocker treatment enhanced bone mass through both enhancement of osteoblastic activity and suppression of osteoclastic activity *in vivo* [23]. Thus, the AT1- and AT2-involved AngII signaling pathway play important roles in regulating bone metabolism.

However, the efficacy of RAS-targeting drugs is often compromised by the reactive renin increase caused by disruption of the renin feedback inhibition, and high renin buildup increases the risk of AngII-dependent and -independent organ damage [24, 25]. Therefore, more alternatives should be considered to block renin directly from the beginning and in combination with the other methods. There are no much studies about the effect of direct renin inhibitors on bone quality, but it can be suggested that these methods may also have similar or better beneficial effects alone or in combination with the other RAS inhibition methods. Aliskiren is active direct renin inhibitor approved for hypertension treatment which has showed a therapeutic potential similar to that of other antagonists of the RAS [26]. More *in vivo* studies may help to exploit the possibility that RAS blockage using aliskiren may have better osteoprotective effects.

### 3.3. Clinical Studies

Further evidence for a potential role of the RAS in bone metabolism as well as the therapeutic effect of RAS inhibition comes from clinical studies. Several studies have compared patients with risk of fractures who have used ACEI and ARBs with patients at similar risks, but no users of these drugs and a significant difference in BMD were recorded [27]. Another separate study also reported that patients treated with an ACE inhibitor showed an increased BMD and more importantly reduced fracture risks [28]. These results imply that RAS inhibitors that are currently being used to treat cardiovascular diseases such as hypertension could be at the same time used for bone disorders which are usually associated with other cardiovascular diseases. 

To achieve more effective blockage of the RAS, different classes of drugs can be used in combination. Aliskiren could effectively block the generation of active renin and of downstream components of the RAS in both nonhypertensive and hypertensive human subjects [25]. In this respect, the direct renin inhibitor differs from the ACE inhibitors and ARBs, which attenuate feedback inhibition of renin synthesis and release by AngII, resulting in a reactive rise in plasma renin activity [25]. This makes it more desirable to conduct further researches on searching molecules that can effectively inhibit renin production and activity. Additional clinical trials can be performed to assess the efficacy and side effects of monotherapeutic and in combination of the drugs since Aliskiren has been recorded to induce weight loss in some hypertensive patients with a mechanism that is not yet understood [29]. 

## 4. Action Mechanism of RAS Inhibitors

**Figure 1:**
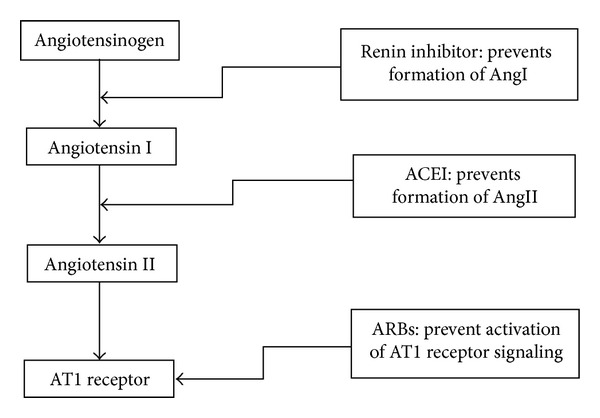
Specific sites for the action of RAS inhibition drugs.

As discussed above, the osteoprotective benefits of inhibiting the RAS are attributed primarily to reduced level of AngII and the activity of its signaling pathway. The overall mechanism by which each type of RAS inhibitor achieves its benefit is described as following (Figure 1).

### 4.1. Renin Inhibitors

In the past, ACE inhibitors and ARBs have been in use for 15–20 years and have proved beneficial effect in reducing AngII level and related disorders. However, these medications were shown to cause renin elevation which has deleterious effect suggesting that it is better to block renin [30]. Renin is a circulatory enzyme secreted by the kidneys, and it acts on angiotensinogen. Renin inhibitors bind to the active site of renin and inhibit its binding to angiotensinogen, which is the rate-determining step of the RAS cascade and consequently prevent the formation of AngI and AngII [31]. Aliskiren is the first-known representative of a new class of completely nonpeptide, orally active, and renin inhibitors and has been shown to inhibit the production of angiotensin I and II [32]. Aliskiren binds to the S3^ bp^ binding site of renin essential for its activity thereby reducing plasma renin activity and suppressing the formation of both AngI and AngII [31, 33]. The efficacy of aliskiren is closely correlated to the fact that renin is a highly specific protease with no other substrate than angiotensinogen and highly species specific [34]. Aliskiren is a nonpeptide, piperidine, designed by molecular modeling of transition-state analogs of angiotensinogen, and it binds with high affinity to the active site of renin [35]. Even though its high molecular weight results in a low bioavailability, the absorbed aliskiren is scarcely metabolized and slowly excreted with a consequently long half-life of 24 to 40 hours [36]. Therefore, Aliskiren offers investigating the potential of blocking the RAS at its rate limiting step.

### 4.2. ACE Inhibitors

ACE is a bivalent dipeptidyl carboxyl metallopeptidase which cleaves the C-terminal dipeptide from AngI and converts it to AngII. ACE inhibitors bind to active site of ACE found in the plasma as well as in the bone tissue and inhibit the action of ACE on AngI. This will decrease the formation of AngII and alter most of its effects which appear to be mediated through the AT1 receptor [37]. ACE inhibitors differ in the chemical structure of their active moieties, in potency, in bioavailability, in plasma half-life, in route of elimination, in their distribution and affinity for tissue-bound ACE, and in whether they are administered as prodrugs. ACE inhibitors may be classified into three groups according to the chemical structure of their active moiety. The first group includes captopril and zofenopril which are sulfhydryl-containing agents. The ACE inhibitors in the second group contain dicarboxylate in their active moiety and include more drugs such as Enalapril, Ramipril, Quinapril, Perindopril, Lisinopril, Benazepril, Imidapril, Zofenopril, and Trandolapril. The only drug in the third group is Fosinopril, and it contains Phosphonate in its structure. The actual mechanism by which ACE inhibitors influence bone mass is not entirely understood, but it is commonly assumed that the therapeutic effect comes from decreased angiotensin II levels. However, long term administration of ACE inhibitors was shown to increase plasma renin level due to the short feedback mechanism associated with the decrease in AngII [38]. The other drawback of ACE inhibitors comes from the fact that ACE is a relatively nonspecific enzyme that has substrates in addition to angiotensin I, and thus, inhibition of ACE may result in accumulation of these substrates [39].

### 4.3. Angiotensin II Receptor Blockers (ARBs)

The angiotensin II receptor blockers (ARBs) represent a newer class of RAS-inhibiting agents which are developed to overcome several deficiencies of ACE inhibitors. Their mechanism of action differs from that of the ACE inhibitors in several ways. ARBs have advantage over ACE inhibitors in selectively inhibiting AngII by competitive antagonism of the AngII receptors. These drugs block the activation of AT1 receptor and reduce the adverse effects of activation of the RAS on bone metabolism [39]. Almost all the known clinical effects including its role in metabolic bone disorder of AngII are mediated by AT1 receptor as it has been well documented experimentally and clinically [40]. Therefore, blocking this receptor will reduce the deteriorating effects of AngII on the bone tissue and the direct effects of the receptor itself on bone metabolism.

In recent years, numerous orally active, selective AT1 receptor antagonists have been synthetized which can effectively block AT1 receptor. Six ARB drugs, namely, Losartan, Valsartan, Irbesartan, Candesartan, Telmisartan, and Eprosartan have been accepted by the US Food and Drug Administration and can be used in the USA and various European countries for the treatment of hypertension. This may show their approval in blocking the RAS with less adverse effects compared to ACE inhibitors.

## 5. Future Developments

In the management of patients with bone metabolic disorders and other cardiovascular diseases, high dose of a single class of RAS inhibitors is often necessary to block the system completely and, hence, to obtain the maximal benefits of blocking the RAS. This will apparently result in a higher exposure to the side effects of this particular class of drugs. In addition, this class of drugs might be effective in blocking a particular target molecule but stimulates another one. In these situations, the combination of the three classes of RAS inhibitors discussed above seems to be attractive to improve the overall blockade of the system. 

It has been advocated that the dual blockade approach of ACE inhibitors and ARBs theoretically should result in improved outcomes in both cardiovascular disease and chronic kidney disease which are usually associated with bone disturbances [41]. Once the better way to effectively block the RAS is established, the actual benefits of RAS inhibition on bone quality can be examined more simply. Studies should also try to compare which combinations among RAS inhibitors are more beneficial for bone disorders and for the other cardiovascular diseases independently. The previous studies which mostly try to relate RAS inhibition with improvement of bone disorder associated with other cardiovascular diseases should also deduce the effects from the perspective of an independent bone disorder. The benefit of RAS inhibition on bone diseases should independently be studied as bone metabolism can be altered due to other causes other than hypertension and kidney failures.

In addition, there is an emerging evidence showing the existence of RAS components in the bone marrow microenvironment [42], and the functional and pharmacological experiments have demonstrated that RAS regulates bone marrow stromal cells, and stem cells, thus involving haematopoiesis and tissue regeneration by progenitor cells [42–44]. Whether there is a role of bone marrow RAS in bone metabolism and there are any interactions of the RAS between bone marrow and bone tissue itself still need to be further investigated.
